# The Dark Side of Female HIV Patient Care: Sexual and Reproductive Health Risks in Pre- and Post-Clinical Treatments

**DOI:** 10.3390/jcm7110402

**Published:** 2018-10-31

**Authors:** Thu Hong Khuat, Thu Trang Do, Van Anh T. Nguyen, Xuan Thai Vu, Phuong Thao T. Nguyen, Kien Tran, Manh Tung Ho, Hong Kong T. Nguyen, Thu Trang Vuong, Viet Phuong La, Quan Hoang Vuong

**Affiliations:** 1 Institute for Social Development Studies (ISDS), Hanoi 100803, Vietnam; hongisds@gmail.com (T.H.K.); ttrang.do@gmail.com (T.T.D.); nguyentva@gmail.com (V.A.T.N.); vuxuanthai160382@gmail.com (X.T.V.); ng.thaonp@gmail.com (P.T.T.N.); trankien@vnu.edu.vn (K.T.); 2 School of Law, Vietnam National University, Hanoi 100803, Vietnam; 3 Centre for Interdisciplinary Social Research, Thanh Tay University, Yen Nghia, Ha Dong, Hanoi 100803, Vietnam; hoang.vuong@thanhtay.edu.vn; 4 Vuong & Associates Co., Hanoi 100803, Vietnam; htn2107@caa.columbia.edu (H.K.T.N.); lvphuong@gmail.com (V.P.L.); 5 Sciences Po, 75337 Paris, France; thutrang.vuong@sciencespo.fr

**Keywords:** HIV care and treatment, women, sexual and reproductive health, Vietnam

## Abstract

This study examines the pre- and post-clinical issues in human immunodeficiency virus (HIV) care and treatment for women and girls of high-risk population groups—namely sex workers, injecting drug users, women living with HIV, primary sexual partners of people living with HIV, adolescent girls who are children of these groups, and migrant young girls and women—in five provinces and cities in Vietnam. Through a sample of 241 surveyed participants and 48 respondents for in-depth interviews and 32 respondents in the focus group discussions, the study identifies multiple barriers that keep these groups from receiving the proper health care that is well within their human rights. Most respondents rated HIV testing as easily accessible, yet only 18.9% of the surveyed women living with HIV disclosed their infection status, while 37.8% gave no information at the most recent prenatal care visit. The level of knowledge and proper practices of sexual and reproductive health (SRH) care also remains limited. Meanwhile, modern birth control methods have yet to be widely adopted among these populations: only 30.7% of respondents reported using condoms when having sex with their husband. This increases the risks of unwanted pregnancy and abortion, as well as vulnerability to sexually transmitted infections (STIs) and HIV transmission. On the other hand, HIV-related stigma and discrimination at health care settings are still pervasive, which create significant barriers for patients to access proper care services. Based on these results, six recommendations to improve SRH status of women and girls of populations at high risk are put forward.

## 1. Introduction

Clinical approaches to the human immunodeficiency virus (HIV), particularly in the developing world where the epidemic persists, consistently highlight the importance of HIV testing as a preventive measure and gateway to the timely initiation of antiretroviral therapy (ART) [1]. This is understandable given that early detection of HIV is proven to prevent mother-to-child transmission [2], and to reduce the overall transmission rate and patient mortality [1,3,4,5,6]. HIV treatment is also increasingly focused on addressing gender inequality in regards to the epidemics, in light of the higher incidence of reported HIV in women than in men [7,8,9,10]. Women and adolescent girls are not only a socially and economically vulnerable population, they are also at higher risks of HIV, sexually transmitted infections (STIs), and other sexual and reproductive health and rights problems [7,11,12]. As such, there is strong support for the implementation of integrated programs of sexual and reproductive health care and STI/HIV/AIDS control [12,13,14].

This study adds to the extant literature by looking at the plight of HIV-infected female patients from Vietnam, offering insights into the limits of clinical treatment and inviting discussion on the need for more comprehensive programs to prevent and fight HIV/AIDS. Vietnam provides an exemplary case study of a populous developing country that has achieved laudable progress in combating HIV/AIDS, but at the same time is struggling to sustain this momentum due to depleting foreign aid [15]. By official accounts, as of April 2015, there were 224,611 people living with HIV in Vietnam [16], putting the HIV prevalence rate at under 0.3% of its population, versus over 36 million HIV-positive people worldwide and the global HIV prevalence rate of 0.8% [17]. Much of this progress is attributed to improvements in the accessibility and quality of HIV/AIDS treatment, care and support services. The treatment coverage in Vietnam, including access to ART, amounts to 67.6%, which is significantly higher than that of the Asia Pacific (53%) and world averages (59%) [16,17]. Yet, the fact that HIV/AIDS is still among the three leading causes of mortality in Vietnam [18] raises a question as to why the availability of medical services remains inadequate in pushing back HIV transmission and mortality.

### 1.1. The Benefits and Shortcomings of Clinical Treatment

This section will review the recommended approach to HIV treatment around the world, in developing countries, and in Vietnam in particular. Considering the remote possibility of an effective HIV vaccine, research on measures to fight and eventually eliminate HIV has invariably stressed the need to apply universal voluntary HIV testing and immediate treatment with ART. One study uses a mathematical model to project that if all HIV-positive adults receive ART for five years, the incidence of HIV and mortality could be reduced to less than one case per 1000 people per year after 10 years of full implementation [1]. Other studies also highlight the immunological benefits of initiating highly active antiretroviral therapy (HAART) at very early stages of chronic HIV-1 infection, including in infected infants, because it allows rapid and almost complete normalization of T cell subsets and preservation of T cell functions [4,5,6,19,20]. The effect of an early-start treatment strategy has also been confirmed in low-income countries [21].

On the adherence to ART in different areas worldwide, a study concludes that differences between the sub-Saharan African and Asia-Pacific regions are attributable to health system resources, although longer ART duration was found to link with better adherence in both places [22,23]. Another meta-analysis pools data from 50 eligible articles from 53 countries and 10,725 patients to find that more than 70% of the HIV-positive adolescent and young adults in Africa and Asia are adherent to therapy while the rates in Europe and North America are lower, at 50–60% [24]. Despite the high rate of adherence to clinical treatment, it is important to note that, as of 2018, the Asia-Pacific region still has the second largest number of HIV-positive individuals after Africa, at 5.2 million people [17]. Because epidemics in Asia are deemed ‘concentrated’ while those in most of the southern and parts of eastern Africa are ‘generalized’ [25,26,27], different approaches are needed. In a qualitative study, HIV-infected patients in India have pointed to the cost of ART as a major barrier, while also noting privacy and stigma issues, such as the disclosure of HIV inhibiting pill-taking and social support [28]. Another study notes the systemic and structural barriers to treatment for injecting drug users (IDUs) in low- and middle-income countries in eastern Europe and Asia, including Vietnam, where this group of patients accounts for the largest share of the HIV-positive population [29]. One of the reasons for this is the fact that many IDUs are either detained or incarcerated in settings where ART and opioid substitution treatment (OST) are unavailable. Overall, the most frequently cited barriers include patients’ lack of information about ART, perceived high costs for ART, and the lack of funding or insurance coverage, plus social stigma, in addition to a lack of integrated services and limited community involvement [23,24,30].

In the case of Vietnam, it was only in 2005 that ART became more widely accessible. The policy, however, has ensured that HIV-infected women should have access at least to HIV testing and nevirapine prophylaxis wherever available, as well as to adequate counselling, HIV infection staging, antiretroviral prophylaxis, and infant formula [31]. By February 2018, 125,806 patients were receiving ART, accounting for 58% of the infected population and significantly higher than the initial figure of 5000 patients recorded in 2005 [32,33]. However, similar to patients in other developing countries, despite the availability and effectiveness of the treatment, Vietnamese people living with HIV also identify stigma as a strong barrier to treatment adherence [34,35]. In other words, people prefer more confidential, better community-supported ways of treatment and care, fearing that even home delivery of ART could increase social stigmatization. There are grounds for such preferences: positive peer support indeed helps improve the quality of life after 12 months among Vietnamese patients under ART presenting at clinical stages 3 and 4 at baseline [36]. Given that ART is life-long and carries a gender-specific impact, it is important to have regular assessments of the quality of life of HIV patients undergoing this treatment [37]. Along this line, since the Vietnamese government began closing compulsory drug detention centers in late 2012 in response to the United Nations’ call, efforts have been made to build a community-based drug treatment program [38]. It is increasingly clear that access to treatment alone will not necessarily reduce the spread of the disease. In addition, studies have shown that, in Vietnam, annual HIV testing and counselling, combined with immediate treatment for key populations, scale-up of methadone maintenance therapy, and condom use, could bring down new infections by 75–81%, as well as lower the cost to approximately $23 million, versus the estimated $77 million it would cost if no integrated method is used [38].

### 1.2. Sexual and Reproductive Health-Related Rights and Care

As discussed above, focusing solely on the provision of ART as a key solution to curbing the HIV epidemic would be insufficient. There are pre- and post-clinical aspects that should be addressed on an equally important footing. This is because the two major factors affecting the rate and magnitude of HIV growth in Asia, including China, Indonesia, and Vietnam, are the size of the sex worker population and the frequency with which commercial sex occurs [39]. Studies have found both male and female sex workers in Vietnam to be at increased risk of acquiring and transmitting HIV and STIs [40,41,42]. Given that unsafe sex is the second most important risk factor to health in terms of disease burden [12,43], the implication, therefore, is clear: public health policies targeting the HIV/AIDS-affected population need to address sexual and reproductive health (SRH) rights and care. Coverage should especially pay attention to high-risk groups, such as men engaging in same-sex intercourse, IDUs, clients and workers involved in commercial sex, adult women, and young adolescents.

Similar to the literature on ART, much of the research on SRH rights and care for HIV-positive people is Africa-centric, even if studies have pointed out the gender-specific impacts of the epidemic. For instance, studies have noted that South African men were more likely than women to fall subject to alcohol abuse and risky sexual behaviors, while women were more likely to suffer from post-traumatic stress disorder [44]. Further, there is an almost equal proportion of South African men and women living with HIV not intending to have children and willing to have children [45,46]. Another study finds a link between sexual trauma and HIV infection among South African women, bringing attention to the need for routine screening for sexual trauma in HIV care settings [47]. A large volume of research on SRH programs in developing countries in general has highlighted the acute risks of STIs and HIV faced by the youth. In a quite comprehensive study that pools data on adolescent reproductive health from three computerized databases (POPLINE, MEDLINE, and ERIC), as well as databases for international health/development organizations, Speizer, et al. [48] conclude that most interventions appear to have had a positive impact on adolescent sexual reproductive health-related knowledge and attitudes. However, this impact was only observed on a minority of the interventions surveyed, and there lacks evidence supporting the efficacy of youth-friendly health services in developing countries [48].

In the case of Vietnam, of the studies that are not related to antiretroviral regimens, scholars have tried to find the correlates of HIV testing among transgender women in Ho Chi Minh City [49] and the link between alcohol abuse and health status of HIV patients receiving treatment [50], as well as to analyze the expenditure for HIV testing and counselling services [51] and clients’ willingness to pay for such services [52]. Some notable findings include: safe sexual behaviors being strongly associated with higher rates of HIV testing [49]; and, female HIV patients with middle-income levels and opioid use are less willing to pay for a voluntary counselling and testing service, compared to those with university-level education [52]. The most recent studies on sexual health promotion in Vietnam appear to focus more on the population of male sex workers or men who have sex with men [40,53,54,55]. This seems to leave a gap for understanding the sexual and reproductive health of the Vietnamese female population affected by HIV.

Within the scope of this study, we note the disturbing rise in the proportion of young women infected and living with HIV—more than half of the HIV-infected young people (15–25 years old) are female [9,10]. Although treatment could allow people living with HIV to have a life expectancy as long as that of the general population, recent large-scale analyses of Canadian HIV-positive individuals have found that female patients have shorter life expectancy and higher mortality rates compared to males [56,57]. Such findings highlight an urgent need for response programs that integrate clinical medicine and comprehensive sexual and reproductive health services targeting female patients of all ages. This paper contributes to research on HIV care in developing countries by providing insights into the pre- and post-clinical issues.

## 2. Methods

### 2.1. Sample and Study Setting

This study is conducted per the request from the Center for Supporting Community Development Initiatives (SCDI) within the component—*Support to adolescent girls and young women (AGYM) most vulnerable to HIV infection*—of the PITCH project (Partnership to Inspire, Transform and Connect the HIV response). The study was approved by the Internal Review Board in Human Subject Research of the Institute for Social Development Studies (Letter of Approval #ISDS-03-2016). Since 2008, the members of the research team have completed the United States National Institutes of Health (NIH) Web-based training course on “Protecting Human Research Participants.” The study was finally approved in the second meeting on 17 October 2016 after reviewing the revised documents.

The research team conducted a survey, in-depth interviews and focus group discussions in a total of five localities in Vietnam, namely, Hanoi, Thai Nguyen, Hai Phong, Nghe An and Ho Chi Minh City. Among these, the three metropolitan areas of Hanoi, Hai Phong, and Ho Chi Minh City were chosen to represent different geographical areas with the largest HIV epidemics in Vietnam. Hanoi, the capital city and populated by over 7 million people, had nearly 20,000 people living with HIV (PLWH) as of late 2017, making up 10% of Vietnam’s total HIV population [58]. Hai Phong, a port city in the northern region where 1.9 million people reside, had 7635 HIV-infected individuals [59]. Ho Chi Minh City, the biggest metropolis with a population of 8.4 million, reported 46,853 HIV patients [60]. For the other two localities, Thai Nguyen marked 6252 cases of HIV infection [61] while Nghe An had 5647 cases [62]. Table 1 summarizes the five locations examined here.

Using the convenience sampling method, the team selected participants by approaching several peer group leaders in five provinces/cities who then introduced the team to their network of members and clients based on a predetermined set of criteria relating to age, occupation, and status of drug using and HIV. The team then contacted these individuals and debriefed them on the study’s objectives. For those who showed an interest in enrolling, the team arranged with peer group leaders to invite them to the groups’ office, where the respondents gave their consent and were either interviewed or selected for a focus group discussion. All activities were conducted in privacy in order to ensure the confidentiality of respondent personal information. For those under 18 years old, the team requested the company of a guardian with whom a consent form was signed prior to each interview.

The mixed methods approach was chosen for the purpose of clarifying and contextualizing the results from both the quantitative and qualitative data analysis. The approach was expected to help us gain both depth of qualitative understanding and the reach of quantitative insights [63,64]. Furthermore, the in-depth interviews allowed for the exploration of viewpoints, beliefs, and experiences of the individual subjects of this study, while the group dynamics in the focus group discussion could generate more diverse feedback and other useful data, such as group languages or narrative [65]. As SRH and HIV treatment are topics loaded with social stigma that can potentially interfere with how the subjects respond, the triangulation method helped this study overcome this limitation and generate insights that were free from the biases of a single method [66].

### 2.2. Survey

The team conducted a survey among women and girls of key populations in five provinces/cities using a semi-structured questionnaire. The questionnaire covered wide-ranging areas related to SRH and HIV treatment and care, and included seven sections: (1) general background; (2) health and reproductive health; (3) miscarriage and abortion; (4) sexual health and birth control; (5) sexually transmitted infections and diseases; (6) sexual health; and (7) experience of sexual abuse.

### 2.3. In-Depth Interviews and Focus Group Discussion

The in-depth interviews (IDIs) explored respondents’ understanding of their SRH rights and HIV/AIDS legislation in five provinces/cities. The interviews elicited perspectives on particular aspects related to access to SRH and HIV care and treatment, as well as their experience of HIV-associated stigma and discrimination at health care centers. Using interview guidelines, the research team interviewed various groups of women and girls, including female sex workers (FSW), primary sexual partners (PSP) of people living with HIV (PLWH), women living with HIV (WLWH), female injecting drug users (IDU), and vulnerable adolescent girls. In addition, interviews were also conducted with health care personnel at both public and private medical institutions to learn about their experiences working with key population groups and to draw from their assessment on these groups’ needs of SRH and HIV care.

In certain areas, focus group discussions (FGD) were held to obtain diverse ideas and perceptions on SRH and HIV rights and access. These discussions were also crucial to gathering participant opinions with regard to their needs and expectations for eliminating HIV-related stigma and discrimination, as well as for improving overall access to SRH and HIV care.

## 3. Results

### 3.1. Desciptive Statistics

In total, the study recruited 241 participants for the survey, 48 respondents for the in-depth interviews, and 32 respondents in the focus group discussions, in five provinces/cities (namely, Hanoi, Thai Nguyen, Hai Phong, Nghe An and Ho Chi Minh City). The mean age of the survey sample is 30.7 years old (min = 15; max = 58). Table 2 describes general information of survey respondents.

The team conducted a total number of 48 in-depth individual interviews and four focus group discussions involving another 32 participants—which brought the total number of participants to 80—across the five aforementioned provinces/cities. Additionally, at the early stage of the research, we also undertook informal discussions with participants at the Vietnam Civil Society Partnership Platform on AIDS (VCSPA) in Ninh Binh; findings from these discussions were also analyzed. Table 3 presents the sample selected for the in-depth study in the five provinces/cities.

### 3.2. Pre- and Post-Clinial Issues

#### 3.2.1. HIV Testing

This study reveals that most respondents possessed quite good knowledge of HIV transmission, prevention and control. Furthermore, HIV testing does not seem to be an issue among the studied populations. Most respondents rated HIV testing as easily accessible, convenient, fast, and reliable. Clinics offering free-of-charge HIV testing services are widely operated with international and local financial support. It is also noteworthy that communication activities enabling HIV testing services to properly reach high-risk populations group have received significant attention. In addition, during in-depth interviews respondents also expressed satisfaction with the confidentiality of personal information when accessing HIV testing centers. However, the fact that respondents were selected through the network of peer groups should also be considered, in the sense that they might have received project support and attended various outreach programs on HIV-related topics and, hence, their situation might not be the general picture of the wider population.

A concern in this area is the insufficient knowledge among adolescent girls, particularly young migrant domestic workers, despite their high risk of exposure. In-depth interviews with this group showed a notable lack of knowledge on HIV or the necessary practices to prevent infection. This might have been a result of limited formal education or the under-coverage of current intervention programs, which often neglect this vulnerable group.

Surprisingly, although HIV testing is rated as accessible, a majority of Vietnamese health care workers are not informed on the HIV status of the respondents. Among the WLWH in this survey, at their most recent prenatal care visit, only 18.9% disclosed their infection status while 37.8% gave no information. Even at the time of labor, a significant percentage of respondents (36.6%) indicated that birth attendants did not know about their HIV status; this proportion is the highest in Hai Phong (71.4%). When HIV-infected respondents had an abortion, around a third reported not to reveal their health status to the health staff. Among respondents who had given birth, only 48.2% were required to undergo HIV testing before going into labor.

#### 3.2.2. Sexual and Reproductive Health-Related Issues

##### Abortion

**Figure 1:**
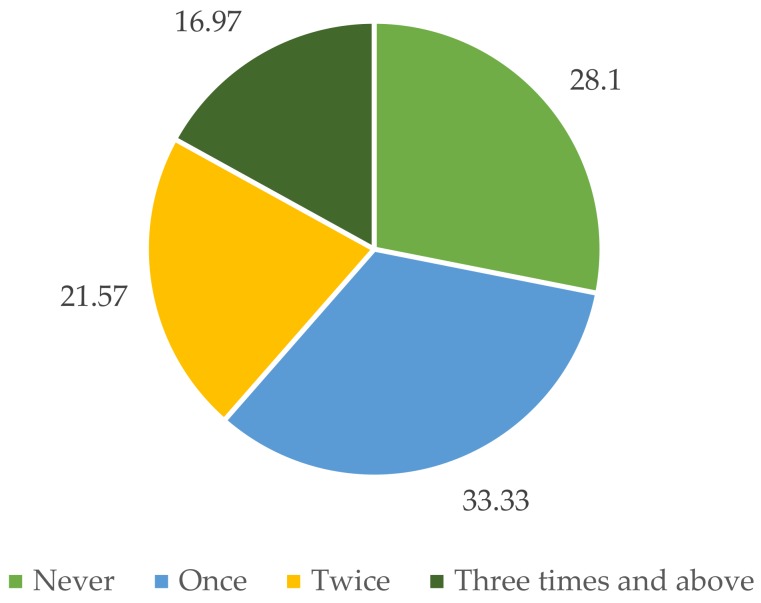
Practice of abortion in five survey locations (%).

According to the survey, as summarized in Figure 1, there is a high prevalence of abortion practice: 71.9% of respondents who have ever been pregnant reported to have had at least one pregnancy ending in abortion. Box 1 presents the viewpoint of the respondents regarding abortion: it appears the at-risk population has accepted abortion, even multiple abortions, as a common practice.

Box 1 **Abortion as a common practice.**

“Young girls who have just started sex work do not have good knowledge of birth control. Most of them have already terminated pregnancy. We regard abortion as a very popular practice.” *(FSWs focus group discussion, VCSPA (Vietnam Civil Society Partnership Platform on AIDS), Ninh Binh)*

“We (high-risk women) usually have infections and sexual health problems. We have too many abortions so easily get infected. Many of us get pregnant easily and continually. Some had abortion last month, this month were pregnant again.” *(Female, 49 years old, in-depth interview, Hai Phong)*

Strikingly, as high as 16.9% of respondents reported to have had at least three abortions. This is perhaps due to the availability of abortion services. Access to abortion services was rated as “very easy” or “easy” by 37.6% and 46.6% of respondents, respectively. Only 4.8% found it difficult to access such services. Similar patterns were witnessed among different regions. The only exception was Thai Nguyen, where 17.1% respondents rated access to abortion services as ‘difficult’.

Concerning the health care setting for certain services, the results show that, although public health facilities remain the most popular point for natal care among respondents in the survey, private clinics were the most popular option for pregnancy termination. In-depth interviews reveal that most respondents opted for private facilities for convenience and because the medical personnel had better attitudes, even though the costs might be higher. Many HIV-infected respondents were more comfortable at such centers because of higher privacy. It was reported that patients were not asked to take an HIV test nor reveal their HIV status before having an abortion. In other words, the HIV status of patients remains completely unknown to these private health facilities. While this brought the patients comfort, it raises deep concerns about the capacity of many private healthcare centers in implementing necessary prevention against HIV transmission from patient to patient, especially when their hygiene protocols could in some case be called in question.

##### Sexual Health

**Figure 2:**
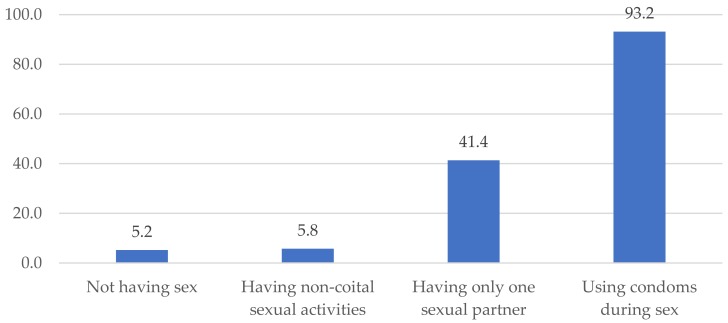
Safe sex behavior (%).

The majority of respondents, 85.3%, reported they understood safe sex practices, with using condoms being the most popular option (Figure 2). The patterns were similar among regions.

Yet, there was a noticeably low prevalence of actual condom use when having sex with a husband, as less than a third of respondents (30.7%) reported doing so in the survey. The corresponding figures were slightly higher at 33% for sexual intercourse with boyfriends/sexual partners and at 48.1% when having sex with others. A significant proportion of respondents cited the purposes of preventing STIs and HIV transmission as their reasons for adoption such methods, besides family planning.

**Figure 3:**
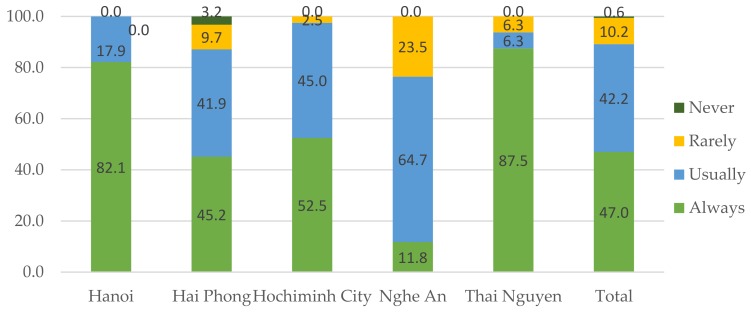
Condom use among FSWs (female sex workers) (%).

The use of condoms among female sex workers was not very high, with only 47% saying “always” and nearly 11% “never or rarely”, and varied across regions (Figure 3). The main reason for not using condom during sex work was attributed to clients’ demand. This situation was cited by 71.6% of FSWs who rarely or never used condoms.

**Figure 4:**
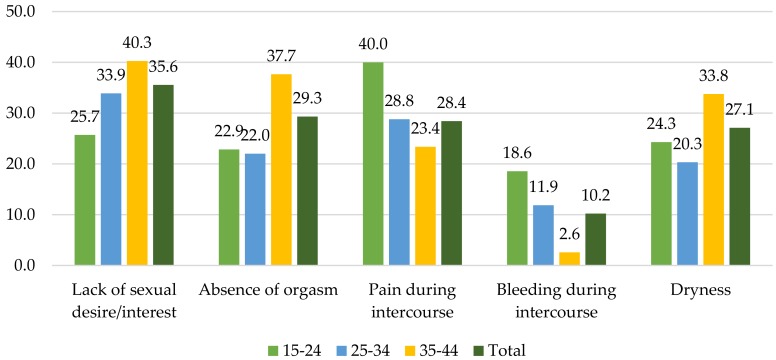
Types of sexual dysfunction (%).

Given that research often shies away from the topic of sexual dysfunction, this study investigated these questions in both the survey and interviews to obtain a more comprehensive picture of the sexual health of the target population. Figure 4 describes five main types of sexual dysfunction that were reported by the respondents. The highest percentage of respondents (35.6%) mentioned lack of sexual desire or interest as the major difficulty they faced. Among other problems are absence of orgasm, pain during intercourse, and dryness, which were cited by 29.3%, 28.4%, and 27.1% of the respondents, respectively. Sexual disorders seemed diverse among different age groups, while lack of sexual desire/interest appeared as the most common problem for women aged 25 to 34 and 35 to 44. The youngest age group mostly cited pain during intercourse as their critical disorder.

To deal with sexual dysfunction, an overwhelming majority (57.0%) reported to leave their problems unattended. Small percentages of those having sexual disorders either purchased medication from pharmacies without prescription, sought advice from friends or counselors, or communicated with their spouses. Only 11.7% went to healthcare clinics to receive treatment and all of these cases opted for private rather than public health centers. In-depth interviews (see Box 2) further confirmed findings from the survey, whereby scant attention was paid to dealing with sexual dysfunctions among respondents despite a fact that the prevalence of those reporting such problems was noteworthy.

Box 2 **Self-treatment of sexual health problems.**

“Whenever I have any symptoms I usually go to pharmacy to buy some medicines. This is more convenient. I am afraid of going to the clinic because I do not want to see a male doctor. I do not want to have the device to be inserted to my vagina either.” *(Female, 39 years old, in-depth interview, Thai Nguyen)*

“I used drugs so I did not have high sexual desires nor want to have sex with my ex-husband. My vagina was always dry. I lost my menstruation quite often. But I did not do anything.” *(Female, 29 years old, in-depth interview, Ho Chi Minh City)*

“After I took antiretroviral drugs, my sexual desires were reduced. But my partner’s desire was still the same. We had less sex than previously. I felt uncomfortable though he did not complain nor force me to please him. I never saw a doctor.” *(Female, 30 years old, in-depth interview, Hanoi)*

##### Physical and Sexual Violence

To gain an understanding of the prevalence of all forms of violence, the study interviewed women and girls of key populations on their experience of physical, emotional and, especially, sexual violence. Overall, nearly one in three respondents (32.3%) reported that they faced physical violence from others during the past 12 months. The prevalence of emotional violence drastically increased to 44.8%, making it the most common form of violence against respondents in the study.

**Figure 5:**
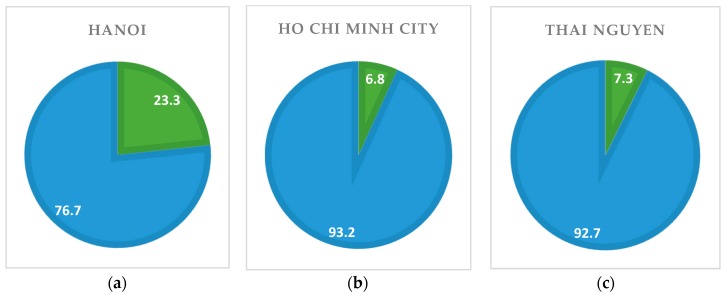

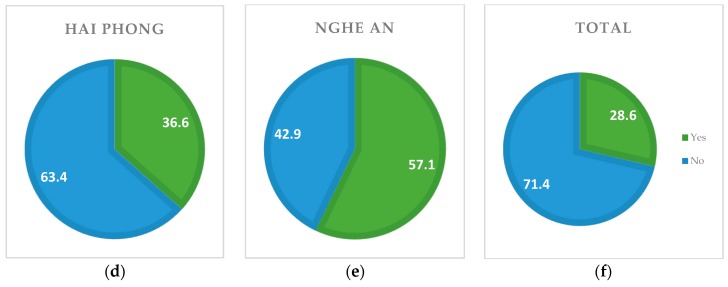
Sexual violence prevalence by localities (%). (**a**) Hanoi; (**b**) Ho Chi Minh City; (**c**) Thai Nguyen; (**d**) Hai Phong; (**e**) Nghe An; (**f**) Total.

With regards to sexual violence, 28.6% said they had suffered from one or multiple types of sexual acts, such as coerced penetration, forced gang sex, sexual harassment, sexual organ torture, and denial of condom use. Figure 5 indicates sexual violence situations in different locations. As can be seen, Nghe An recorded the highest proportion of sexual violence victims, with 57.1% of respondents, followed by Hai Phong (36.6%). Meanwhile, Ho Chi Minh City and Thai Nguyen revealed substantially lower incidence, relatively at 6.8% and 7.3%.

**Figure 6:**
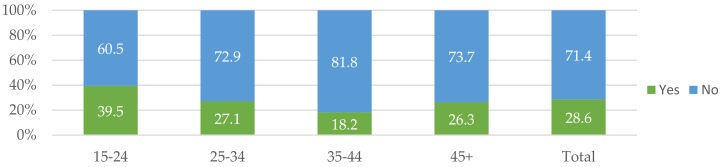
Sexual violence by age groups (%).

By age group, sexual violence prevailed the most among the youngest surveyed populations. Figure 6 suggests that 39.5% of respondents in the 15-to-25-year-old age group suffered from sexual violence during that past 12 months prior to the survey, which is notably higher than for the overall sample, and more than double the figure for the group aged 35 to 44, which recorded the lowest prevalence.

In terms of violence perpetrators, clients were cited by 60% of the survivors of general physical violence. Intimate partners were also quite common, as 27.2% of the survivors mentioned their husband and 31.2% pointed to their boyfriend or primary sexual partner as the perpetrators. Other violence perpetrators include employers, pimps, co-workers, family members, and law enforcers such as police officers.

In addition, 74.3% of the surveyed FSW reported to have suffered from violence caused by their clients. Some other perpetrators who were mentioned as inflicting violence on the FSWs included family members, brothel owners, sex brokers, and co-workers. Physical violence was perpetrated on 49.2% of the FSW respondents, while the prevalence of emotional and sexual violence was 72.9% and 55.1%, respectively. The three most common types of sexual violence committed by clients were coerced sex, rape threats, and sexual harassment.

It is worth noting that in-depth interviews suggest that FSW working in organized brothels seem less likely to fall victim to violence from clients. These sex workers usually operate in certain places, such as designated hotels or guest houses, which are known to their managers and colleagues, who provide assistance and protection in case of emergency. Meanwhile, independent FSWs lack all support in sex work and face increased risks of all forms of violence. Box 3 presents the respondents’ experience of different types of sexual violence.

Box 3 **Different types of sexual violence.**

“One of the women working with me once met a client. He had abnormal preference. He asked my friend to let him sit on her shoulder and pee. After that he took off his belt and used it to beat her.” *(Female, 49 years old, in-depth interview, Ho Chi Minh City)*

“I used to experience violence several times. For instance, I had a client. I asked him to wear condoms during sex but he refused. And he said bad words to me. He cursed and shouted at me. If I could not convince him, he would hit me.” “A young woman working with me, recently she had a client. He used drug. He paid her VND200,000 in advance. They had sex but the guy could not ejaculate. He went angry and hit her. He also called his friends to bring a knife to the hostel and threaten to kill her if she did not pay the money back.” *(Female, 49 years old, in-depth interview, Hai Phong)*

“The restaurant I work at opens all night. There are many drunk customers. They often make some dirty jokes and make fun of me. Most of the time I could not do anything. I did not know what to do.” *(Female, 17 years old, in-depth interview, Hanoi)*

“I was caught by the police once. One man told me to have sex with him and after that he would let me go without paying a fine.” *(Female, FSW focus group discussion, Ho Chi Minh City)*

### 3.3. Non-Clinical Issues

#### 3.3.1. Services at Healthcare Settings

Pervasive stigma and discrimination can take many forms but can be classified into two broad categories: attitudinal and behavioral. Common forms of attitudinal manifestations include verbal harassment, gossip, or taking excessive protection measures. Other discriminatory practices from health care providers include neglect, denial of services, differential treatment, and disclosure of HIV status without the patients’ consent. In particular, participants in the study pointed out prenatal care visits and labor time as the situations with the worst and most pervasive stigma and discrimination. Interviews and group discussions with these target groups suggest that health care workers tended to use protective measures in an inappropriate way while attending to HIV patients for SRH or HIV treatment, such as wearing another layer of mask, gloves or goggles. Box 4 presents the detailed descriptions of the discrimination faced by the respondents.

Box 4 **Experience of HIV-related stigma and discrimination.**

“I have a client who has HIV. She went to an outpatient clinic for obtaining a treatment request. The staff there asked her to sit in a far distance. She was shocked and did not want to receive the treatment anymore.”

“A woman with HIV I know on the day she gave birth the staff at hospital talked to her: “Why do you still give birth when you have already contracted with AIDS?”. She was made to leave the hospital right after her partum. They did not allow her to stay.”

“I went to have a pelvic test. I told the doctor that I am HIV infected. Then the doctor asked me to open my vagina on my own for her to insert the device (speculum). She (the doctor) did not want to touch my vagina. I felt very embarrassed.” *(FSWs focus group discussion, VCSPA, Ninh Binh)*

“If they (healthcare workers) saw any trace of drug injection on my body they changed their attitudes right away. They did not care if I have HIV or not. They put on gloves and mask.”

“Doctors and nurses, they often whispered and gossiped to each other. When I had an ultrasound scan, the physician used a separate towel. When I went to have gynecological test they often tell each other to be careful with me.” *(Female, IDUs Focus group discussion, VCSPA)*

“I went to the city dermatology hospital (A national specialized hospital) and told the doctors that I am a person with HIV and currently under ARV treatment. Immediately, they wore another layer of glove and did not look at me. Both the female and male doctors showed their discriminatory attitudes. Every time I had that experience.” *(Female, 44 years old, in-depth interview, Ho Chi Minh City)*

“Doctors are now more sensitive when seeing sex workers. Even when they know about our status they do not show any discrimination. But receptionist at health clinics still do.” *(Female, 43 years old, in-depth interview, Ho Chi Minh City)*

“I delivered at Hung Vuong hospital [a public hospital]. After the HIV testing the doctors found out that I am positive. They changed their behaviors immediately. After I gave birth, they did not intend to shower my baby. I recalled one day there was an intern. She did not know about my status and held my baby for me. But another one came in and told the intern (about my HIV status). And she refrained from touching my baby though my baby does not have HIV. It was different from the first time I gave birth, also at this hospital.” *(Female, FSW focus group discussion, Ho Chi Minh City)*

The in-depth study also involved healthcare workers in both public and private settings to learn about the access of key populations to SRH and HIV care, as well as to assess the needs of high-risk groups in different aspects regarding health care provision. All visited health care centers have received support from donor-funded HIV/AIDS prevention and control projects at some point in time. The team met with staff who have had direct contact with patients of high-risk populations, for instance, testing physicians or doctors.

Findings from the interview and observation at respondents’ offices showed that most health care centers were well-equipped with adequate facilities, infrastructure and preventive measures against the transmission of contagious diseases. Many respondents had been actively taking part in collaborative activities community-based organizations and peer groups such as mobile clinics, sensitization programs and communication campaigns at different locations.

However, the interviews with these health care providers also reflect several limitations that hinder the effectiveness of their work in delivering care and treatment to the key populations. First, most centers continue to face human resource constraints, which frequently puts current staff under excessive workloads. This potentially means that service quality may fall short of patients’ expectations. Second, most respondents reported a lack of refresher training, particularly relating to the necessary skills to work with people of high-risk populations. Training courses mostly focus on technical aspects and are not held on an annual or semi-annual basis, which means health care workers have little chance to review and update their knowledge. Finally, dependence on project support remains quite widespread, implying significant concern regarding the sustainability of these centers upon the termination of donors’ funding.

#### 3.3.2. Health Insurance

Access to and use of health insurance remains a key concern for the community of HIV at-risk populations in Vietnam. The research revealed several constraints that limit the surveyed population from accessing and using their health insurance. Most commonly, it is because the individual did not put much importance on health insurance, or was unaware of the administrative procedure to purchase insurance. The possession of health insurance varied from group to group. Most WLWH participating in the study reported having a health insurance that covers ART and other HIV treatment. This group often expressed good awareness of the necessity for being covered by health insurance and reported frequent use of their insurance cards.

Younger women were generally not aware of the importance of having health insurance. The common reason was that these respondents had never before, at least at the time of the study, experienced any complicated health problems and seldom visited the hospital, so they did not regard health insurance as important. In addition, lack of information on the procedure for purchasing health insurance was also cited as a factor that hindered the willingness of studied participants to buy and use health insurance. The under-coverage of HIV-related costs in existing insurance schemes was quite frequently mentioned as a factor discouraging women in this study from obtaining and using insurance cards.

Migrant female domestic workers shared difficulties in getting health insurance because having a permanent residence is a required condition for health insurance purchase. Regulation of household insurance schemes, which is still in effect in some regions, also imposed a notable barrier for women and girls purchasing health insurance, especially those of low-income families.

## 4. Discussion

### 4.1. Limitations

The most significant limitation of this study is the sampling method. As the respondents were selected through a network of peer groups, the results presented in this study should be interpreted with caution. For example, the respondents might have received support from HIV-related outreach programs, and their views can be influenced by these programs. As noted by the authors of [67,68], the convenience sampling method is suitable to apply for hard-to-reach populations, but might not be suitable for generalizing to a larger population. However, as five population centers throughout Vietnam are represented in this study, this limitation might have been compensated for. In addition, given the sample size of 241 participants, in which various demographic characteristics are represented, further correlation studies can be performed.

### 4.2. Policy Implications

This study shows that there is a severe stigma in health care settings against PLHIV and key populations. It is especially worrying that to avoid stigma, many HIV patients may not disclose their HIV status while health workers not always strictly practice universal precaution when providing services of abortion or labor. Answers from the respondents also show their greater comfort with private facilities, because in these facilities they are less stigmatized than in public facilities. The problems are clear: public healthcare centers are often understaffed; current medical personnel still lack specific training to deal with high-risk key populations; and the training for health workers on how to work with key population quite significantly depends on international funding. The results suggest policies that increase training for healthcare workers in this area, as suggested by [69], and this should also cover private healthcare centers on HIV-related matters.

Another notable result is the pervasive stigma and discrimination in healthcare services towards HIV patients. As other studies have shown, stigma can prevent optimal results in clinical treatments [70,71,72]; the finding suggests intervention programs targeting HIV-related social stigma to further increase the effectiveness of ART. There have been successful attempts of such initiatives on an institutional level in the past [73]. While that is an encouraging sign, there seems to be little effort at implementation on a national scale [74].

Regarding participant attitude towards health insurance, this study reveals that awareness of the importance of health insurance and knowledge of the administrative procedures to purchase health insurance are two constraints that limit the access and use of the service. As people are shown to be very sensitive to the cost of health care service [75,76], being covered by health insurance can positively impact the treatment of HIV. Yet, in this study, the under-coverage of HIV-related costs in existing insurance schemes is often cited among the leading factors discouraging women from buying and using health insurance. Such a finding not only calls for a need to further include HIV care and treatment in health insurance programs, which Vietnam has been undertaking since 2008, but also for more activities to increase public awareness of these programs, as well as for insurance system reforms, as suggested by [77].

With specific regard to adolescent girls, during in-depth interviews this group often showed reluctance discussing sexuality-related issues partly because most adolescent respondents had little to no sexual experience or formal sex education. On the other hand, it also reflects that adolescents are poorly informed and lack adequate access to information on SRH. It was shown that adolescent girls in this survey tended to rely on their peers and friends rather than their parents or teachers when seeking information or counseling on sexuality and reproductive health matters. Meanwhile, the role of school education on such issues was rarely mentioned. This finding carries high implications for their health and overall well-being and highlights the need for parents, educational institutions and interested stakeholders to provide adolescents with accurate information so as to make them well-prepared for any sex-related decisions.

Overall, the study provides an updated take on the sexual and reproductive health of Vietnamese women vulnerable to HIV infection as research exploring this topic appears quite outdated [31,73,78,79,80,81]. The findings confirm once again the need for more effective programs that improve the quality of health clinics and reproductive health campaigns, as well as the attitude of healthcare workers. In a broader context, given that Vietnam is facing international donor withdrawal in the HIV/AIDS sector, funding for ART will be cut short in the next few years [15], which would drive a higher need for an integrated clinical approach to treating the disease. In particular, the funding problem is alarming as the Global Fund to Fight AIDS, Malaria, and Tuberculosis and the United States’ President’s Emergency Plan for AIDS Relief (PEPFAR) programs, which supported 80% of Vietnam’s HIV/AID response in 2008–2013, are planning to exit the country entirely [15]. Indeed other studies have verified the declining trend in donor governments’ funding for HIV in low- and middle-income countries: $7.53 billion in 2015 compared to $8.62 billion in 2014 [82,83]. As such, the study makes a convincing case for other developing countries facing a similar funding shortage to also look for comprehensive solutions in dealing with HIV patients and the at-risk population.

## 5. Conclusions

This study of women and girls of high-risk population groups in five provinces and cities of Hanoi, Thai Nguyen, Hai Phong, Nghe An, and Ho Chi Minh City examines pre- and post-clinical issues in HIV care and treatment. Although various policies and national programs, as well as interventions targeting these groups, have led to improvements in SRH and HIV care and treatment in recent years, the study identifies multiple barriers that constrain these groups from attaining proper care and enjoyment of their health rights. Most notably, knowledge and proper practices of SRH care remains limited. Negligence and self-treatment of SRH health risks are quite common. Another problem is the unpopular adoption of modern birth control methods, which increases the risks of unwanted pregnancy and abortion as well as vulnerability to STIs and HIV transmission. On the other hand, HIV-related stigma and discrimination remain pervasive in health care treatment settings, which create significant barriers for patients to access proper care. To improve the SRH status of women and girls of populations at high risk, the study proposes six recommendations, namely: (i) increase awareness among policymakers and lawmakers on SRH rights and relevant legal frameworks; (ii) promote communication activities on health insurance; (iii) enhance STI services for women and girls of high-risk groups as part of any HIV control strategy; (iv) boost skill training and capacity building for healthcare workers; (v) implement rigorous measures to identify and assist victims of sexual abuse; and, (vi) prioritize interventions targeting adolescent girls.

## Figures and Tables

**Table 1:** Summary of study settings. The number of people living with HIV (PLWH) by geographical locations.

	Geographical Location	Total Population	Number of PLWH (End-2017)	Percentage of Vietnam’s PLWH Population
Hanoi	Northern	7.65 million	19,904	9.5%
Thai Nguyen	Northern	1.15 million	6252	3.0%
Hai Phong	Northeastern	1.96 million	7635	3.6%
Nghe An	North-central	3.11 million	5647	2.7%
Ho Chi Minh City	Southern	8.43 million	46,853	22.4%

**Table 2:** Background characteristics of survey respondents.

	Number of Respondents	Percentage
*Location*		
Hanoi	30	12.4%
Hai Phong	41	17.0%
Ho Chi Minh City	59	24.5%
Nghe An	70	29.0%
Thai Nguyen	41	17.0%
*Age group*		
15–24	86	35.7%
25–34	59	24.5%
35–44	77	32.0%
45 and above	19	7.9%
*Marital status*		
Married	55	22.8%
Widowed	21	8.7%
Divorced	40	16.6%
Separate	7	2.9%
Cohabiting	13	5.4%
Never married	105	43.6%
*Highest educational level*		
Primary school	45	18.7%
Lower secondary school	101	41.9%
Upper secondary school	93	38.6%
Vocational training-college-university	2	0.8%
Total	241	

**Table 3:** In-depth study sample. (FSW: female sex workers; WLHIV: women living with HIV; PSP of PLHIV/IDU: primary sexual partners of people living with HIV/injecting drug users).

	Hanoi	Hai Phong	Nghe An	Thai Nguyen	Ho Chi Minh City	Total
*In-depth interview*						48
FSW	1	1	1	1	1	5
WLHIV	1	1	1	1	1	5
PSP of PLHIV/IDU	2	2	2	2	2	10
IDU woman	1	1	1	1	1	5
Woman 3 in 1 *	1	1	1	1	1	5
Adolescent girl	3	1	2	1	1	8
Health care worker	2	2	2	2	2	10
*Focus group discussion*						4
Adolescent girl						
FSW					1	1
FSW and IDU woman					1	1
PSP of PLHIV/IDU		1				1
WLHIV				1		1

*: FSW, IDU and HIV.
